# Evaluation of Efficacy of Bromocriptine as a Therapeutic Modality in the Treatment of Diabetes Mellitus: A Systematic Review

**DOI:** 10.7759/cureus.41931

**Published:** 2023-07-15

**Authors:** Priyansh Patel, Amulya Adusumilli, Dharaneswari Hari Narayanan, Diya Patel, Sunny Patel, Sai Dheeraj Gutlapalli, Sunil Patel, Kavan G Patel, Borislav Kheyson, Suzy Bibawy, Philip Otterbeck

**Affiliations:** 1 Department of Internal Medicine, California Institute of Behavioral Neurosciences & Psychology, Fairfield, USA; 2 Department of Internal Medicine, Medical College Baroda, Vadodara, IND; 3 Department of Internal Medicine, Mahadevappa Rampure Medical College, Kalaburagi, IND; 4 Department of Internal Medicine, Government Medical College Omandurar Government Estate, Chennai, IND; 5 Department of Internal Medicine, Gujarat Medical Education and Research Society, Sola, Ahmedabad, IND; 6 Department of Internal Medicine, St. George's University School of Medicine, St. George's, GRD; 7 Department of Internal Medicine, Richmond University Medical Center, New York, USA

**Keywords:** bromocriptine mesylate, cycloset, diabetes mellitus (dm), drug therapeutics, dopamine agonist

## Abstract

Diabetes mellitus (DM), one of the oldest diseases known to mankind has always been difficult to treat even with the availability of a variety of medications. In such a scenario, the Food and Drug Administration (FDA) has approved a novel therapeutic, bromocriptine, with a different mechanism of action than the traditional medications since 2009 but has not been used as either first-line therapy or add-on therapy. In this systematic review, we searched databases like PubMed, Medline, PubMed Central, Cochrane Library, Clinicaltrials.gov, and Wiley Online Library. The selected articles were screened using inclusion and exclusion criteria and quality appraised; finally, 11 studies including eight clinical trials and three narrative reviews were included. It was found that an increase in dopamine and serotonin levels were hypothesized to convert the insulin-resistant (IR) state to an insulin-sensitive (IS) state. Hence in DM, as there is an IR state, the administration of dopamine was hypothesized to increase insulin sensitivity. In our study based on included studies, it was found that bromocriptine was superior as an add-on therapy to metformin compared to metformin alone, also it was found beneficial in people failing treatment with any one oral hypoglycemic agent. On the contrary, bromocriptine was found inferior to teneligliptin in treating DM. Still, more studies are required to make an accurate and reliable assessment of the efficacy of bromocriptine in treating DM.

## Introduction and background

Diabetes mellitus (DM) has been recognized as one of the most ancient diseases in human history. Egyptians described diabetes in their ancient texts around 3000 years ago. The term "diabetes" originates from the Greek language specifically from the word "siphon" which was coined by Araetus of Cappadocia (81-133 AD) and signifies "to pass through." Due to the characteristic sweetness imparted to the urine and blood of patients with diabetes, the Latin term "mellitus" (sweet) was added by Thomas Willis in 1675 [1,2]. From ancient times to the current era, DM is still one of the most commonly occurring diseases affecting around 476 million people in over 90 countries out of which more than 33% of people are unaware of their disease status [3,4]. It also accounts for about 450,000 deaths in the United States (US) each year [4]. DM belongs to a group of metabolic disorders that occurs due to defects in insulin secretion, insulin action, or both which cause the blood glucose to stay elevated pathologically [2,5]. According to the previous classification, DM was classified as insulin-dependent DM (IDDM) and non-insulin-dependent DM (NIDDM) but later due to the identification of other types of DM which did not fit into this classification, it was reclassified where IDDM was classified as type 1, and NIDDM was classified as type 2, and other specific types [6]. The primary components of therapy for type 2 DM involve the use of various medications, such as sulfonylureas, biguanides, thiazolidinediones, meglitinides, α-glucosidase inhibitors, glucagon-like peptide-1 (GLP-1) receptor agonists, dipeptidyl peptidase-4 (DPP-4) inhibitors, and amylin agonists. Bromocriptine and colesevelam are effective, albeit rarely used treatment options for DM globally, although there have been studies that have shown the effectiveness of bromocriptine as a treatment option for DM [7,8]. While bromocriptine was approved by the Food and Drug Administration (FDA) for DM in 2009 as an adjunct to diet and exercise [7], it is still infrequently used both as first-line therapy and as add-on therapy later in the disease course. There can be myriad reasons for this including the complexity of the titration schedule, pill burden, side effect profile, high product cost, and general lack of familiarity with the product. Furthermore, the mechanism by which bromocriptine acts has also not been fully understood. In this systematic review, we plan to evaluate the mechanism of action, efficacy, and safety of bromocriptine in patients with DM as monotherapy and compare its efficacy as an add-on therapy with some of the first-line treatments for DM like metformin, sulfonylureas, and teneligliptin.

## Review

Methodology

Preferred reporting items for systematic reviews and meta-analysis (PRISMA) 2020 guidelines were used for conducting this systematic review [9].

Search Strategies

Databases like PubMed, Medline, PubMed Central, Cochrane Library, ClinicalTrials.gov, and Wiley Online Library were used to search for various combinations of bromocriptine and DM. However, the following strategy was developed and used to search PubMed’s MeSH database for more relevant literature: ("Diabetes Mellitus/drug therapy"[Majr] OR "Diabetes Mellitus/therapy"[Majr]) AND ("Bromocriptine/therapeutic use"[Majr]). Table 1 shows the databases used with their corresponding search strategies and keywords along with the number of papers identified using each strategy.

**Table 1:** Databases with corresponding search strategies and studies identified PMC: PubMed Central

Database	Search strategy	Papers identified	Full text
PubMed, PMC, Medline	Diabetes Mellitus AND Bromocriptine	190	54
	("Diabetes Mellitus/drug therapy"[Majr] OR "Diabetes Mellitus/therapy"[Majr]) AND ("Bromocriptine/therapeutic use"[Majr])	42	12
Cochrane Library	Diabetes Mellitus AND Bromocriptine	50	49
ClinicalTrials.gov	Diabetes Mellitus AND Bromocriptine	9	7
Wiley Online Library	Diabetes Mellitus AND Bromocriptine	1153	23
Total	-	1444	146

Eligibility Criteria

We selected literature published in the English language with full text. All types of studies like clinical trials, observational studies, qualitative studies, quantitative studies, review articles, case studies, and experimental studies, from all times were included. Papers focusing on the glucose-lowering effects of bromocriptine in diabetic patients were included with and without other anti-diabetic drugs. Studies involving only human participants of all age groups were included. Articles were excluded where the full text could not be retrieved. Also, articles evaluating effects other than glucose-related effects were excluded. Gray literature and proposal papers were also excluded. Table 2 shows all inclusion and exclusion criteria.

**Table 2:** Inclusion and exclusion criteria CVS: Cardiovascular

Inclusion criteria	Exclusion criteria
Papers written and published in the English language	Papers other than the English language
Papers discussing glucose-related effects of bromocriptine in diabetes	Papers showing effects on CVS or renal system (other than glucose related)
Papers comparing glucose-reducing effects of bromocriptine in adjunct to other anti-diabetogenic drugs	Trials whose results are not retrieved
Papers include mixed types of studies	Papers where the full text cannot be retrieved
Papers are included from all times	Gray literature and proposal papers
Papers only include full texts	-
Papers focusing on all age groups	-

Selection Process

Each identified article was extracted and screened. All identified duplicates were removed. All remaining articles were screened through titles and abstracts. In case of a conflict about eligibility, the concerns were discussed with all other co-authors and finalized by mutual consensus. The selected articles were critically evaluated by assessing for quality using quality appraisal tools. Only relevant articles satisfying inclusion and exclusion criteria were shortlisted.

Quality Appraisal of the Studies

The shortlisted articles were checked for quality using relevant quality appraisal tools. The evaluation was done and a mutual consensus was finalized in case of a potential conflict. Cochrane risk-of-bias 2 (RoB-2) assessment tool was used for randomized control trials. Narrative reviews were assessed using the scale for the assessment of narrative review articles (SANRA) checklist. Only the studies that passed the quality checks were included in this systematic review. Tables 3, 4 show the quality appraisal tools used for corresponding shortlisted studies.

**Table 3:** Cochrane risk-of-bias 2 (ROB2) assessment tool DM: Diabetes mellitus, BCQR: Bromocriptine quick release, GLP: Glucagon-like peptide

No.	Clinical trials	Bias of randomization process	Effect of assignment to intervention	Effect of adhering to intervention	Bias due to missing outcome data	Bias in the measurement of outcome	Bias in selection of reported result
1.	Safety and tolerability study of Cycloset in treatment of type 2 DM [10]	Low risk	Low risk	Low risk	Medium risk	Low risk	Low risk
2.	Efficacy and safety of add-on therapy of bromocriptine with metformin in Indian patients with type 2 DM: a randomized open-labeled phase IV clinical trial [11]	Low risk	Low risk	Low risk	Low risk	Low risk	Low risk
3.	BCQR as adjunct therapy in type 1 DM [12]	Low risk	Low risk	Low risk	Medium risk	Low risk	Low risk
4.	Effect of Cycloset on glycemic control when added to GLP-1 analog therapy [13]	High risk	Low risk	Low risk	Medium risk	Low risk	Low risk
5.	BCQR as an adjunct to insulin and metformin in the treatment of type 2 DM [14]	High risk	Low risk	Low risk	Low risk	Low risk	Low risk
6.	Evaluation of the efficacy and safety of BCQR in type 2 DM [15]	Low risk	Low risk	Low risk	Medium risk	Low risk	Low risk
7.	Assessment of safety and efficacy of bromocriptine in comparison with teneligliptin in newly diagnosed type 2 DM [16]	High risk	Low risk	Low risk	Low risk	Low risk	Low risk
8.	Bromocriptine: a novel approach to the treatment of type 2 DM [17]	Low risk	Low risk	Low risk	Low risk	Low risk	Low risk

**Table 4:** Scale for assessment of narrative reviews DM: Diabetes mellitus

No.	Reviews	Justification of article’s importance for readership	Statement of concrete/specific aims or formulation of questions	Description of literature search	Referencing	Scientific reasoning	Appropriate presentation of data
1.	Bromocriptine: A sympatholytic, D2-dopamine agonist for the treatment of type 2 DM [18]	2	2	0	2	2	2
2.	Bromocriptine in type 2 DM [19]	2	1	0	2	2	2
3.	Bromocriptine approved as the first medication to target dopamine activity to improve glycemic control in patients with type 2 DM [20]	2	2	0	2	2	2

Data Collection Process

Data was extracted and all co-authors were equally involved in finalizing the retrieved data. All shortlisted articles were critically reviewed for primary outcomes like change in glucose levels and change in hemoglobin A1c (HbA1c) and secondary outcomes like change in fasting plasma glucose (FPG) and change in postprandial plasma glucose (PPPG). All the outcomes were showing effects on glucose like change in glucose levels, change in HbA1c, change in FPG, and change in PPPG.

Results

Study Identification and Selection

**Figure 1:**
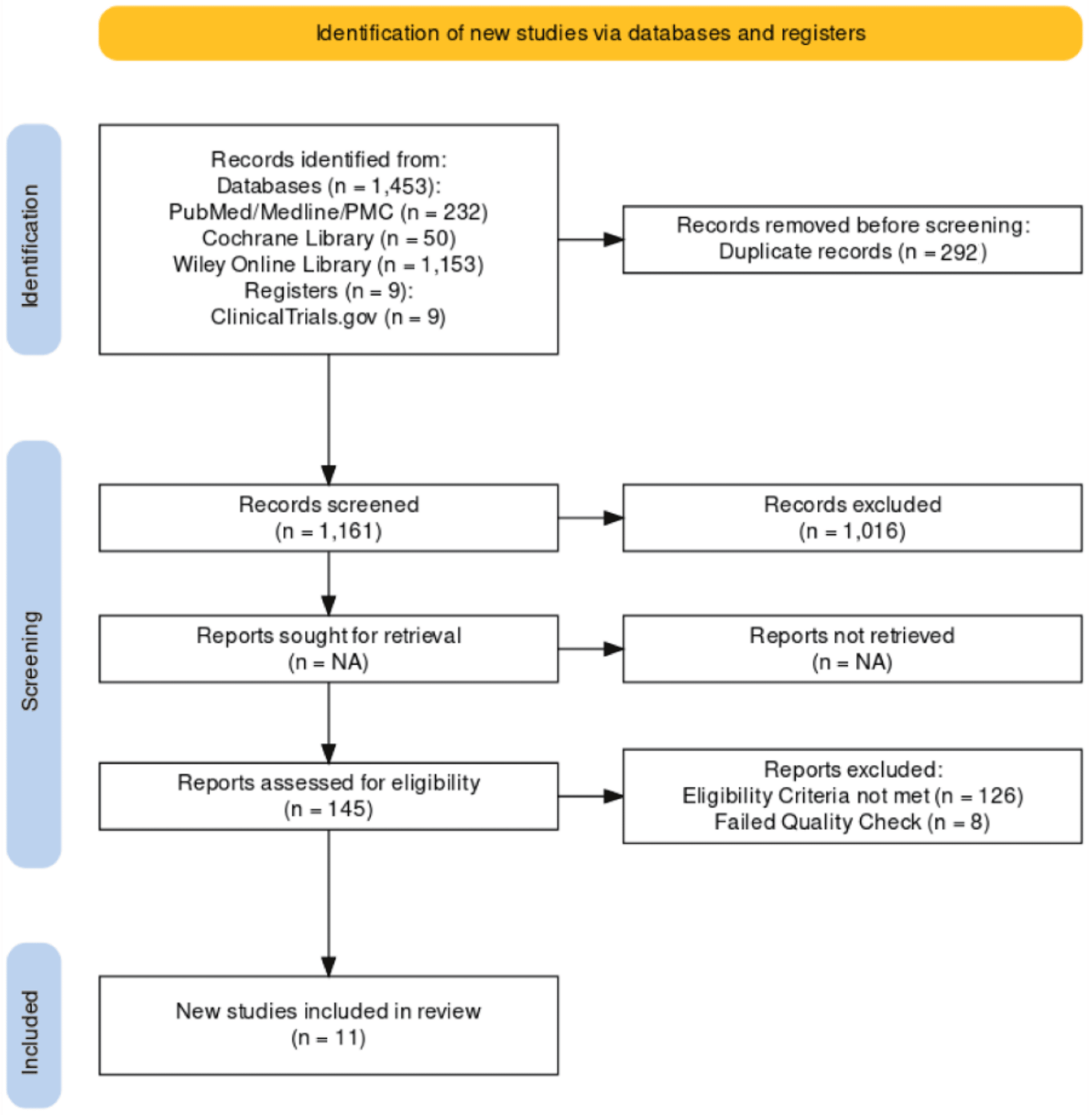
PRISMA flowchart PMC: PubMed Central

A total of 1453 articles were identified using relevant databases and registries. Out of them, 292 were removed as duplicates before the screening process. Then screening the remaining articles based on abstracts, titles, retrieving full texts, inclusion, and exclusion criteria, we were left with a shortlist of 11 papers, out of which eight were randomized control trials and three were narrative reviews. The selection process is shown in Figure 1 as a PRISMA flowchart [21].

Discussion

DM is a chronic metabolic disorder of multifactorial origin characterized by markedly elevated blood sugar, polyuria, polydipsia, polyphagia [5,18,19]. The pathogenesis of DM ranges from autoimmune destruction of pancreatic beta cells that produces insulin thereby causing insulin deficiency to disorders that cause insulin resistance (IR) and multiple other pathophysiologic defects [19]. IR is caused by an overabundance of fatty acids and proinflammatory cytokines, leading to impaired glucose transportation and elevated fat breakdown. As a result of insufficient insulin response or production, the body compensates by inappropriately increasing the production of glucagon, exacerbating hyperglycemia [2].

Classification

**Figure 2:**
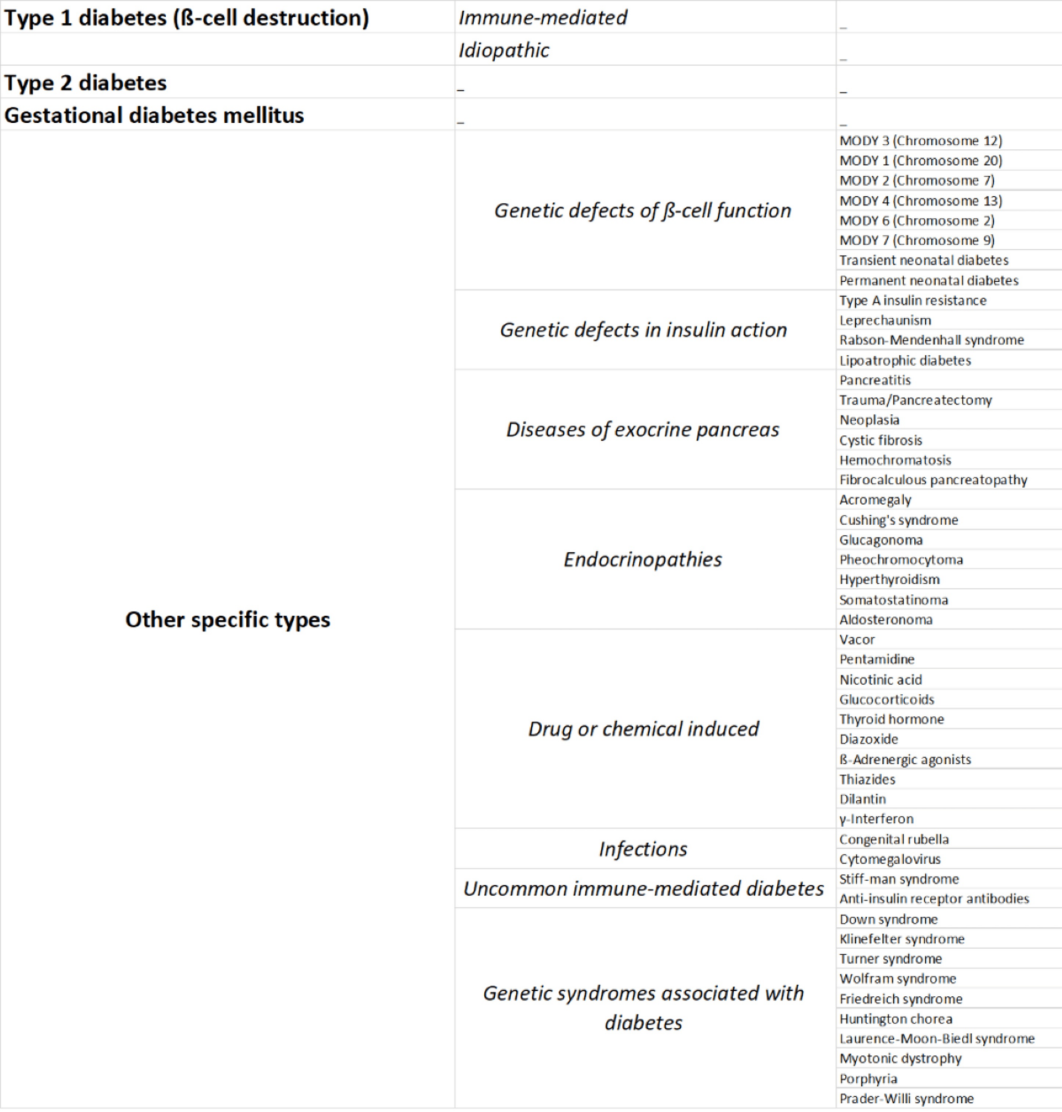
Classification of diabetes mellitus MODY: Maturity onset diabetes of young

The different etiologies involved in the pathogenesis of DM are shown in Figure 2 [5].

Complications

Persistently elevated blood glucose can lead to broad and deleterious effects, resulting in both microvascular and macrovascular complications. Microvascular complications include nephropathy, neuropathy, and retinopathy whereas macrovascular complications include atherosclerotic cardiovascular disease (ASCVD) with vessel narrowing most commonly in the heart, extremities, carotid arteries, and the brain. ASCVD is considered one of the most dreadful complications of DM [2]. The severity of all the complications of DM is dependent on the time of uncontrolled hyperglycemia, thus early diagnosis and treatment are very essential in preventing complications of DM [2,5].

Diagnosis

According to American Diabetes Association (ADA), DM can be diagnosed by using biomarkers like FPG, two-hour plasma glucose, random plasma glucose (RPG), and HbA1C. Off these currently, HbA1C is the most accurate and widely used biomarker for diagnosis of DM because it reflects average blood glucose levels over 120 days as it does not fluctuate with change in everyday blood glucose levels and stays relatively constant over 120 days and hence correlates well with both microvascular and macrovascular complications [5]. Any one criterion mentioned in Table 5 is sufficient to make the diagnosis of DM [5].

**Table 5:** Diagnostic criteria for diabetes mellitus HbA1C: Hemoglobin A1C, FPG: Fasting plasma glucose, RPG: Random plasma glucose, OGTT: Oral glucose tolerance test, mg/dL: milligrams per deciliter, mmol/L: millimoles per liter, g: grams

Test	Diagnostic values	Remarks
HbA1C	Greater than or equal to 6.5%	5.55% - 6.4% is known as prediabetes
FPG	Greater than or equal to 126 mg/dL (7.0 mmol/L)	Fasting is defined as no intake of calories for at least 8 hours
Two-hour plasma glucose during OGTT	Greater than or equal to 200 mg/dL (11.1 mmol/L)	The test should be done using equivalent of 75 g anhydrous glucose dissolved in water
RPG	Greater than or equal to 200 mg/dL (11.1 mmol/L)	RPG is measured in patients with classic symptoms of hyperglycemia

Treatment

Insulin analogs: Insulin analogs are extremely effective in reducing blood glucose levels but they were found to be limited in their ability to reproduce physiologic insulin secretion patterns. Hence, using insulin, especially with both short and long-acting formulations results in a significant risk of symptomatic and severe hypoglycemia. Insulin analogs are of different types depending on the onset of action; rapid-acting (insulin lispro, aspart, and glulisine), intermediate-acting (neutral protamine hagedorn (NPH) insulin, lente insulin, ultralente insulin), long-acting (insulin glargine, detemir, degludec) [8].

Non-insulin medications: Current non-insulin medications include sulfonylureas, acts by blocking potassium adenosine triphosphate (ATP)-dependent channels thereby increasing insulin secretion (glyburide, glipizide, and glimeperide); biguanides, acts by improving peripheral glucose uptake and inhibiting gluconeogenesis (metformin, phenformin); meglitinides, acts by insulin secretion by binding to SUR1 receptors on pancreas (repaglinide, nateglinide); thiazolidinediones, acts by binding to perixosome proliferator activator receptors which in-turn increases insulin sensitivity (pioglitazone, rosiglitazone); GLP-1 analogs acts by stimulating glucose dependent insulin secretion as well as insulin synthesis while simultaneously inhibiting glucagon release (exenatide, liraglutide, semaglutide, dulaglutide, lixisenatide, and tirzepatide); dipeptidyl peptidase (DPP)-4 inhibitors, acts by inhibiting the enzyme dipeptidyl peptidase thereby increasing the levels of GLP (sitagliptin, saxagliptan, linagliptin, and alogliptin); sodium glucose co-transporter-2 (SGLT-2) inhibitors, helps by eliminating glucose via urine (canagliflozin, dapagliflozin, empagliflozin, ertugliflozin); alpha-glucosidase inhibitors acts by reducing absorption of glucose from gastrointestinal tract (acarbose, voglibose, and miglitol); amylin analogs, bromocriptine, colesevelam. With the exception of sulfonylureas and insulin, these drugs lower sugar in a glucose-dependent manner. Even though FDA approved since 2009, bromocriptine is still not used as a mainstay of treatment for DM, maybe due to the lack of evidence showing its efficacy as an anti-diabetic agent or lack of its side effect profile in diabetic patients [7,8,18].

Bromocriptine, Mechanism of Action in DM

Bromocriptine is a sympatholytic, postsynaptic dopamine receptor agonist and serotonin modulator derived from ergot alkaloid. It is primarily used in the treatment of Parkinson's disease (PD) via dopamine receptors in the nigrostriatal tract and also used in the treatment of acromegaly and hyperprolactinemia via the tuberoinfundibular pathway [20]. DM is considered a risk factor for PD. It has been noted that with the onset of DM in patients with PD there is an increase in the severity of symptoms of PD; even though dopamine does not have action on specific receptor acting of blood glucose and lipid metabolism, this suggests that the onset of DM might exaggerate the effect of dopamine deficiency in PD. This, even as its actions on dopamine receptors to affect blood glucose is still not known [22-25]. In the presence of adequate food sources, mammals tend to have a metabolic insulin-sensitive (IS)/glucose-tolerant phase and during hunger or long periods of food scarcity, the metabolism turns to insulin-resistant (IR)/glucose-intolerant phase for longer survival. In the absence of food, mammals can adapt their metabolism from IS phase to IR phase [18]. Shivprasad et al. showed that there is an increase in lipolytic activity during the IR phase; this leads to increased lipid oxidation by peripheral tissues and reduces glucose utilization [19]. This adaptation causes a rise in gluconeogenesis and hepatic glucose production to increase glucose availability to the central nervous system (CNS) during prolonged periods of hunger or food scarcity [19]. Other animal studies have shown increased levels of serotonin and noradrenaline during the IR state while the activity levels return to normal during IS state while undergoing seasonal changes in metabolism. On the contrary, during the IR phase, there is a decrease in dopamine levels which increase to normal with a return to IS phase [18,19]. In vertebrates these changes in seasonal metabolism in monoaminergic activity are controlled by temporal interaction of circadian neuroendocrine rhythms which occurs in suprachiasmatic nuclei (SCN) and ventromedial hypothalamus (VMH), these are also known as mammalian circadian pacemaker [19]. Via et al. showed that hepatic glucose production via sympathetic pathways is regulated by CNS, it also integrates hormonal signals through leptin, ghrelin, insulin, GLP-1 [20]. Via et al., also hypothesized that since anti-dopaminergic activity by antipsychotic medications causes weight gain, insulin resistance, dyslipidemia, and impaired metabolism, so use of dopamine agonists can be used for reversing IR [20].

Qualitative Analysis of Clinical Trials

We found eight clinical trials during a literature search evaluating the efficacy of bromocriptine, comparing bromocriptine with other current first-line medications. A detailed assessment of all randomized control trails is shown below in Table 6.

**Table 6:** Qualitative analysis of clinical trials DM: Diabetes mellitus, BCQR: Bromocriptine quick release, HbA1c: Hemoglobin A1c, FPG: Fasting plasma glucose, PPPG: Post-prandial plasma glucose, GLP-1: Glucagon-like peptide-1, mg/dL: milligrams per decilitre

No.	Clinical trials	Type of study	Participants involved	Intervention	Control	Duration	Outcomes measured		Sponsor
1.	Safety and tolerability study of Cycloset in treatment of type 2 DM [10]	Randomized control trial-parallel group assignment.	3095	Cycloset (Bromocriptine mesylate): 0.8 mg, 1 tablet/day starting at week 1 and increasing at a rate of 1 tablet/week, up to a target dose level of 6 tablets/day. (2054 subjects)	Placebo (1016 subjects)	52 weeks	Change in HbA1c in subjects failing treatment with metformin and sulfonylurea	Cycloset is significantly superior in reducing HbA1c than placebo	VeroScience
							Change in HbA1c in subjects taking at least one oral hypoglycemic agent	Cycloset is significantly superior in reducing HbA1c than placebo	
2.	Efficacy and safety of add-on therapy of bromocriptine with metformin in Indian patients with type 2 DM: A randomized open-labeled phase IV clinical trial [11]	Randomized controlled trial-parallel group assignment.	74	Group A: Metformin + bromocriptine: 0.8 mg tablet once daily for 3 months. (25 subjects) Group B: Metformin + bromocriptine: 1.6 mg tablet once daily for 3 months (26 subjects) (Metformin is 500 mg twice daily for 3 months)	Group C: Metformin: 500 mg tablet twice daily for 3 months (23 subjects)	20 weeks	Change in HbA1c	The bromocriptine group showed a statistically significant dose-dependent reduction in HbA1c in adjunct to metformin. Group A: 0.89, Group B: 1.32, Group C: 0.37	Dr. Nilanjan Sengupta, Department of Endocrinology Nilratan Sircar Medical College, Kolkata
							Change in FPG (in mg/dL)	Bromocriptine showed a statistically significant dose-dependent reduction in FPG in adjunct to metformin. Group A: 60.3, Group B: 76, Group C: 41.1	
							Change in PPPG (mg/dL)	Bromocriptine showed a statistically significant dose-dependent reduction in PPPG in adjunct to metformin. Group A: 78.9, Group B: 100.4, Group C: 57.6	
3.	BCQR as adjunct therapy in type 1 DM [12]	Randomized control trial-crossover assignment	108	BCQR for 4 weeks then placebo for 4 weeks (43 subjects)	Placebo for 4 weeks then BCQR for 4 weeks (41 subjects)	8 weeks	Glucose levels at the end of 4 weeks	Bromocriptine was significantly superior to placebo in adults	University of Colorado, Denver
4.	Effect of Cycloset on glycemic control when added to GLP-1 analog therapy [13]	Randomized control trial-single group assignment	23	Cycloset (Bromocriptine mesylate): 2.4-3.2 mg/day as tolerated	-	16-20 weeks	Mean change in HbA1c in subjects taking exenatide once weekly or liraglutide	Bromocriptine caused a statistically significant reduction of 0.6 in HbA1c (from 8.3 to 7.7)	The University of Texas Health Science Center at San Antonio
5.	BCQR as an adjunct to insulin and metformin in the treatment of type 2 DM [14]	Randomized control trial-parallel group assignment	15	Group A: BCQR + metformin + Insulin: 0.8 mg (1 tablet/day starting at week 1 and increasing at a rate of 1 tablet/week, up to a target dose level of 6 tablets/day. (10 subjects) (B+M+I group)	Group B: Metformin + Insulin (5 subjects) (M+I group).	24 weeks	Change in HbA1c	Bromocriptine group showed a statistically significantly higher reduction in HbA1c with end-point HbA1c of: Group A: 7.98, Group B: 9.74	University of Texas Southwestern Medical Center
6.	Evaluation of the efficacy and safety of BCQR in type 2 DM [15]	Randomized control trial-parallel group assignment	105	Group A: BCQR 2.4 mg once daily (35 subjects). Group B: Metformin 500 mg twice daily (35 subjects)	Group C: BCQR 2.4 mg once daily + metformin 500 mg twice daily (35 subjects)	12 weeks	Change in mean HbA1C	Bromocriptine showed to be significantly more effective in adjunct to metformin in reducing HbA1C. Group A: 0.46, Group B: 0.63, Group C: 0.74	-
							Change in mean FPG (mg/dL)	Bromocriptine showed statistically significant reductions in FPG in adjunct with metformin. Group A: 16.09, Group B: 37.36, Group C: 44.31	
							Change in mean PPPG (mg/dL)	Bromocriptine showed statistically significant reductions in PPPG in adjunct with metformin. Group A: 14.38, Group B: 35.87, Group C: 43.71	
7.	Assessment of safety and efficacy of bromocriptine in comparison with teneligliptin in newly diagnosed type 2 DM [16]	Randomized control trial-parallel group assignment	50	Group A: Bromocriptine: 0.8-1.6 mg tablet once daily as tolerated	Group B: Teneligliptin: 20 mg tablet once daily	12 weeks	Change in mean HbA1C	Teneligliptin showed a statistically significant reduction in HbA1C compared to bromocriptine. Group A: 0.8, Group B: 1.3	-
							Change in mean FPG (mg/dL)	Teneligliptin showed a statistically significant reduction in FPG compared to bromocriptine. Group A: 30, Group B: 64.36	
							Change in mean PPPG (mg/dL)	Teneligliptin showed a statistically significant reduction in PPPG compared to bromocriptine. Group A: 31.88, Group B: 92.68	
8.	Bromocriptine: a novel approach to the treatment of type 2 DM [17]	Randomized control trial-parallel group assignment	22	BCQR: 0.8 mg, 1 tablet/day starting at week 1 and increasing at a rate of 1 tablet/week, up to a target dose level of 6 tablets/day (15 subjects)	Placebo (7 subjects)	16 weeks	Change in HbA1C	Bromocriptine showed a statistically significant reduction of 0.6 in HbA1C compared to a rise of 0.6 with placebo	-
							Change in FPG (mg/dL)	Bromocriptine showed a statistically significant reduction of 18 compared to a rise of 36 with placebo	

Limitations

The study has some constraints that need to be considered. One limitation is the insufficient presence of high-quality evidence, such as randomized controlled trials or meta-analyses. The analysis was based on a restricted selection of clinical trials that were accessible for analysis. Moreover, there was variability among the studies in terms of sample sizes and the variables that were measured. Certain studies included in the analysis did not evaluate comparable variables or secondary outcomes. Additionally, only papers published in English were included in the review, meaning that information from papers published in other languages was not considered. Also, studies including the effects of bromocriptine on other systems like cardiovascular and renal were not included. Hence further assessment can be done in diabetics with those effects.

## Conclusions

There was a clinical improvement in reducing HbA1C with a progressive increase in the once-daily dosage of bromocriptine quick release (BCQR) in treatment-naive DM patients. Administration of Cycloset (Bromocriptine mesylate) in patients failing treatment with sulfonylurea, metformin, and at least one oral hypoglycemic agent, showed significant improvement in HbA1c with a progressive increase in once-daily dosage levels of cycloset. BCQR was also found to improve blood glucose levels at the end of 4 weeks. There were benefits of bromocriptine as an add-on therapy to metformin with significant improvement in HbA1c, FPG, and PPPG when compared to metformin alone. On the contrary, there was a superiority of BCQR formulation as an adjunct to metformin and insulin in reducing HbA1c. Bromocriptine was found inferior to teneligliptin in treatment-naive DM patients. The results indicated that teneligliptin demonstrated superiority in reducing HbA1c, FPG, and PPPG levels. Still, further research in the form of clinical trials with the observation of long-term effects and a larger sample size is necessary to evaluate the efficacy of bromocriptine as a therapeutic modality for DM.
