# Correction: Evaluation of Efficacy of Bromocriptine as a Therapeutic Modality in the Treatment of Diabetes Mellitus: A Systematic Review

**DOI:** 10.7759/cureus.c308

**Published:** 2025-09-23

**Authors:** Priyansh Patel, Amulya Adusumilli, Dharaneswari Hari Narayanan, Diya Patel, Sunny Patel, Sai Dheeraj Gutlapalli, Sunil Patel, Kavan G Patel, Borislav Kheyson, Suzy Bibawy, Philip Otterbeck

**Affiliations:** 1 Department of Internal Medicine, California Institute of Behavioral Neurosciences & Psychology, Fairfield, USA; 2 Department of Internal Medicine, Medical College Baroda, Vadodara, IND; 3 Department of Internal Medicine, Mahadevappa Rampure Medical College, Kalaburagi, IND; 4 Department of Internal Medicine, Government Medical College Omandurar Government Estate, Chennai, IND; 5 Department of Internal Medicine, Gujarat Medical Education and Research Society, Sola, Ahmedabad, IND; 6 Department of Internal Medicine, St. George's University School of Medicine, St. George's, GRD; 7 Department of Internal Medicine, Richmond University Medical Center, New York, USA

This article has been corrected to fix multiple typos related to the number of articles involved in the search. The PRISMA diagram and first paragraph of the Results section have been corrected as follows:

- Total articles identified: 1,444 corrected to 1,453
- Removed as duplicates: 282 corrected to 292
- Shortlisted: 12 corrected to 11
- Randomized control trials: 9 corrected to 8

